# The roles of interoceptive sensitivity and metacognitive interoception in panic

**DOI:** 10.1186/s12993-015-0058-8

**Published:** 2015-04-08

**Authors:** Adrián Yoris, Sol Esteves, Blas Couto, Margherita Melloni, Rafael Kichic, Marcelo Cetkovich, Roberto Favaloro, Jason Moser, Facundo Manes, Agustin Ibanez, Lucas Sedeño

**Affiliations:** Laboratory of Experimental Psychology and Neuroscience (LPEN), INECO (Institute of Cognitive Neurology) and Institute of Neuroscience, Favaloro, Favaloro University, Pacheco de Melo 1860, Buenos Aires, C1078AAI Argentina; UDP-INECO Foundation Core on Neuroscience (UIFCoN), Diego Portales University, Santiago, Chile; Anxiety and Trauma Clinic, INECO (Institute of Cognitive Neurology), C1078AAI Buenos Aires, Argentina; National Scientific and Technical Research Council (CONICET), Buenos Aires, Argentina; Department of Psychology, Michigan State University, East Lansing, MI USA; Universidad Autónoma del Caribe, Barranquilla, Colombia; Australian Research Council (ACR) Centre of Excellence in Cognition and its Disorders, Macquarie University, NSW 2109 Sydney, Australia

**Keywords:** Anxiety disorder, Panic attacks, Interoception sensitivity, Metacognitive interoception, Heartbeat detection

## Abstract

**Background:**

Interoception refers to the ability to sense body signals. Two interoceptive dimensions have been recently proposed: (a) interoceptive sensitivity (IS) –objective accuracy in detecting internal bodily sensations (e.g., heartbeat, breathing)–; and (b) metacognitive interoception (MI) –explicit beliefs and worries about one’s own interoceptive sensitivity and internal sensations. Current models of panic assume a possible influence of interoception on the development of panic attacks. Hypervigilance to body symptoms is one of the most characteristic manifestations of panic disorders. Some explanations propose that patients have abnormal IS, whereas other accounts suggest that misinterpretations or catastrophic beliefs play a pivotal role in the development of their psychopathology. Our goal was to evaluate these theoretical proposals by examining whether patients differed from controls in IS, MI, or both. Twenty-one anxiety disorders patients with panic attacks and 13 healthy controls completed a behavioral measure of IS motor heartbeat detection (HBD) and two questionnaires measuring MI.

**Findings:**

Patients did not differ from controls in IS. However, significant differences were found in MI measures. Patients presented increased worries in their beliefs about somatic sensations compared to controls. These results reflect a discrepancy between direct body sensing (IS) and reflexive thoughts about body states (MI).

**Conclusion:**

Our findings support the idea that hypervigilance to body symptoms is not necessarily a bottom-up dispositional tendency (where patients are hypersensitive about bodily signals), but rather a metacognitive process related to threatening beliefs about body/somatic sensations.

**Electronic supplementary material:**

The online version of this article (doi:10.1186/s12993-015-0058-8) contains supplementary material, which is available to authorized users.

## Background

Interoception (the ability to perceive bodily sensations) [1] has been proposed as a risk factor for panic attacks [1,2]. Two of its multiple dimensions [3] are related to panic [2]: i) interoceptive sensitivity (IS) –the objective detection of visceral sensations, assessed via tasks such as heartbeat detection (HBD)–, and ii) metacognitive interoception (MI), defined here as participants’ reflexive beliefs and thoughts about one’s own body sensations. While MI has been restricted to explicit knowledge about accuracy during interoceptive tasks [3], we characterize it as beliefs about bodily sensations at large [4].

IS studies in panic disorders are inconclusive [5], with patients performing either better than [2] or similar to [6] controls (Additional file 1: 1.1). These studies have employed two types of HBD tasks: i) mental tracking paradigms, currently questioned because its working memory demands might affect cardiac perception [7]; and ii) discrimination tasks, where interference generated by simultaneous attention to cardiac sensation and external stimuli may constitute a confound [7]. The possibility that these lurking variables may be the source of discrepant results calls for more robust methods in IS research.

MI is consistent with cognitive models of panic which emphasize the misinterpretation of somatic sensations as a fundamental aspect of its psychopathogenesis [8], with patients reporting more worries about body signals than controls [6,9,10].

IS and MI constitute different interoceptive processes [3] which are not necessarily associated [9,10]. Here, we assessed IS through a novel resting HBD paradigm that addresses certain limitations of other resting cardiac IS tasks [11-13] (see its advantages in Additional file 1: 2.2). MI was examined with self-report measures about body sensation beliefs.

Our overarching hypothesis was that patients and controls would differ in IS and MI. Specifically, we predicted that patients, relative to controls, would perform better in IS and obtain higher scores in MI associated with catastrophic beliefs about body signals.

## Methods

### Subjects

The sample compressed twenty-one anxiety disorder patients [14] who experienced at least one panic attack (PA) [2,10] in the month before testing, and 13 healthy controls. Both groups were matched for age, gender, and education (Table 1). The PA group encompassed different DSM-IV anxiety diagnoses, including panic disorder. We selected this broad-range of diagnoses to assess the underlying mechanisms of panic attacks in anxiety disorders patients. In addition, it has been shown that panic attack episodes are similar to panic attacks in panic disorders [15].

**Table 1:** **Demographic, neuropsychological and clinical results**

	**F**	**P**	**Patients**	**Control sample**
*Gender*	0.03 (χ2)	0.85	Male = 12; Female = 9	Male = 7; Female = 6
*Age (years)*	0.00	0.97	M = 32.33; SD = 10.23	M = 32.46; SD = 10.01
*Formal education (years)*	1.20	0.28	M = 15.24; SD = 2.02	M = 16; SD = 1.87
*Body mass index*	2.29	0.14	M = 23.56; SD = 3.28	M = 21.80; SD = 3.12
*Panic Disorder as primary diagnostic*	--	--	13 subjects	--
*Others Anxiety diagnostics.*	--	--	SP (6), SeP (1) and GAD (1)	--
*Mixed diagnostic*	--	--	1 subject (PA and PTSD).	--
*Total of Panic Attacks (last 12 months)*	--	--	M = 6.0; SD = 7.90	--
*Total medication sample (%)* ^*#*^	--	--	47%	--
*BDI-II*	11.29	<0.01*	M = 15.80; SD = 11.53	M = 4.23; SD = 5.54
*STAI Trait*	15.78	<0.01*	M = 47.19; SD = 12.24	M = 32.38; SD = 6.92
*STAI State*	2.36	0.13	M = 34.14; SD = 8.93	M = 29.92; SD = 4.19
*BSQ*	42.74	<0.01*	M = 47.26; SD = 10.72	M = 23.38; SD = 9.22
*PCI*	23.79	<0.01*	M = 2.07; SD = 0.12	M = 1.15; SD = 0.14

*indicates significant differences between patients and controls. M = mean; SD = standard deviation.

SP = social phobia; SeP = specific phobia; GAD = general anxiety disorders; PA = panic disorder; PTSD = post-traumatic stress disorder.

# Medication details are listed in Additional file 1: 3.7.

Patients’ diagnoses were established with the SCID-I [16] by an anxiety disorder expert, and the presence of panic attacks was established according to Barlow’s criteria [17]. Controls had never experienced panic attacks and had no history of drug abuse or neuropsychiatric disease. Body mass index was controlled given its influence on IS [18]. Participants provided informed consent in accordance with the Declaration of Helsinki. The study was approved by INECO institutional ethics committee.

### Mood and anxiety measurements

Mood and depression levels were assessed via the Beck Depression Inventory Second Edition (BDI-II), while state and trait anxiety levels were examined through the State Trait Anxiety Inventory (STAI) (Table 2).

**Table 2:** **Detailed description of self-report questionnaires used**

**The Beck Depression Inventory-II**	(BDI-II) is a 21-item depression scale that assesses emotional, behavioral, and somatic symptoms. Items on the BDI-II are rated on a four-category Likert scale that goes from 0 to 3, with a maximum total score of 63. Higher scores indicate more severe depressive symptoms.
**The State-Trait Inventory**	(STAI) is a 40 item scale, which assesses both state and trait anxiety and represents a well-validated and reliable self-report measure of dispositional and state anxiety. The scales for trait and state anxiety are made up of 20 items. Participants are asked to indicate to what degree the items describe their dispositional and situational feelings on a four-point Likert-type scale.
**Body Sensations Questionnaire**	(BSQ) is a 17-items scale concerning the degree to which patients fear somatic symptoms commonly associated to panic (i.e. dizziness, heart palpitation, chest pressure). Items are related on five point scales regarding from *not frightened or worried by this sensation to extremely frightened by this sensation.*
**Physical Concern Index**	Is a subscale of the Agoraphobic Cognitions Questionnaire (ACQ). It describes thoughts and believes about fear to physical symptoms of anxiety and panic attack.

### IS: Heartbeat Detection Task (HBD)

**Figure 1:**
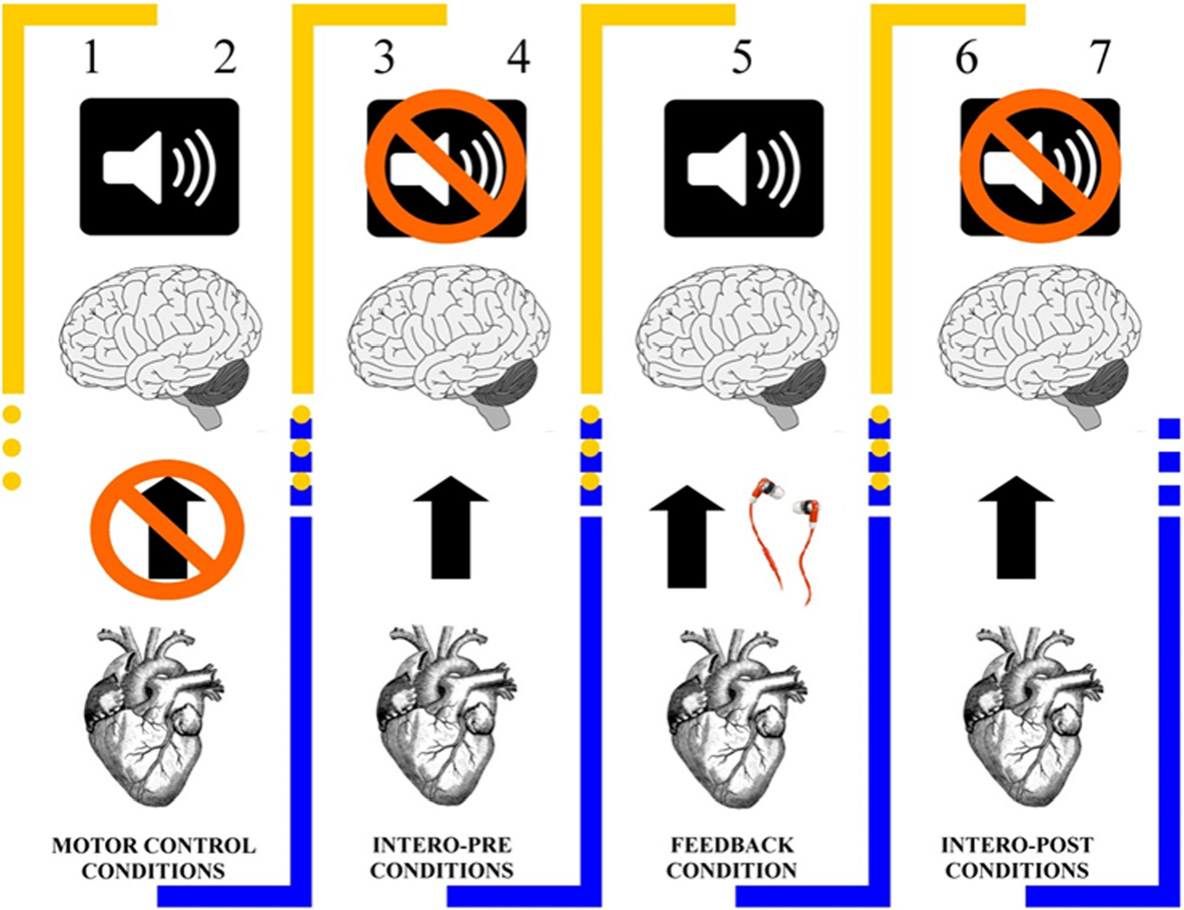
**Experimental design of heartbeat detection task (HBD).** The HBD task, a motor tracking test, is an experimental procedure in which participants tap a keyboard along with their heartbeats in different conditions (each lasting 2 minutes). First, as motor-control conditions, participants followed an audio-recording of a synchronic heartbeat (1) and then a non-synchronic heartbeat (2). Next, they followed their heartbeats without external feedback (intero-pre conditions) in two intervals (3 & 4). Then, in a feedback control condition, they did the same while receiving simultaneous auditory feedback of their own heart provided through online EKG register (feedback condition), (5). Finally, they followed their own heartbeats without feedback (intero-post conditions) two times (6 & 7). These conditions offer a measure of audio-motoric performance (first and second conditions), and a cardiac interoceptive measure prior to (intero-pre condition) and after (intero-post condition) the feedback condition. During this task we also measured heart rate (HR) and heart rate variability (HRV) to control their possible influence on IS (details in Additional file 1: 3.5).

The HBD task is a motor tracking test that assesses IS at rest [11-13]. Participants had to tap a key on a keyboard in rhythm with their heartbeat in different conditions (see Figure 1 and Additional file 1: 2.1 for a detailed explanation).

### MI: self-report questionnaires

Beliefs about body signals were assessed with the Body Sensations Questionnaire (BSQ) and the Physical Concern Index (PCI) of the Agoraphobic Cognitions Questionnaire (ACQ) [19]. Created to target the *fear of fear* construct, these instruments were here used as an index of catastrophic thoughts about interoceptive sensations [6]. The BSQ measures fear of bodily sensations associated with high arousal and panic. The PCI assesses reflexive thoughts about physical concerns and their negative consequences (Table 2).

### Data analysis

ANOVA tests were used for demographic and clinical questionnaires. Categorical variables (e.g., gender) were analyzed with the Pearson chi-square (χ2) test. Mixed repeated measured ANOVAs were performed for HBD, with a within-subject factor (the seven conditions) and a between-subject factor (the two groups; see Additional file 1: 3.1). Considering the possible influence of depression and anxiety on interoception [5,20] − and the significant differences between groups (Table 1)−, we performed an ANCOVA using BDI and STAI (trait and state) scores as covariates. This analysis was applied only to interoceptive conditions from the HBD task and to self-questionnaires of MI. Effect sizes were reported with partial eta (η_p_^2^).

## Results

### Demographic results

No group differences were found in gender [*χ2*(1, 34) *=* 0.03*, p =* 0.85], age [*F*(1, 32) < 0.01*), p =* 0.97*, η*_*p*_^*2*^ *=* 0.04], formal education [*F*(1, 32) *=* 1.20*, p =* 0.28*, η*_*p*_^*2*^ *=* 0.03] or body mass index [*F*(1, 30) *=* 2.29*, p =* 0.14*, η*_*p*_^*2*^ *=* 0.07].

### Clinical results

Group differences for BDI-II [*F*(1, 31) = 11.29*, p* < 0.01*, η*_*p*_^*2*^ *=* 0.26] revealed higher scores of depressive symptoms in PAs than in controls. Both groups showed similar state anxiety levels [*F*(1, 31) *=* 2.36*, p =* 0.14*, η*_*p*_^*2*^ = 0.07]. However, patients exhibited significant higher trait anxiety levels [*F*(1, 32) = 15.76*, p* < 0.01*, η*_*p*_^*2*^ *=* 0.33].

### Interoceptive sensitivity (IS)

**Figure 2:**
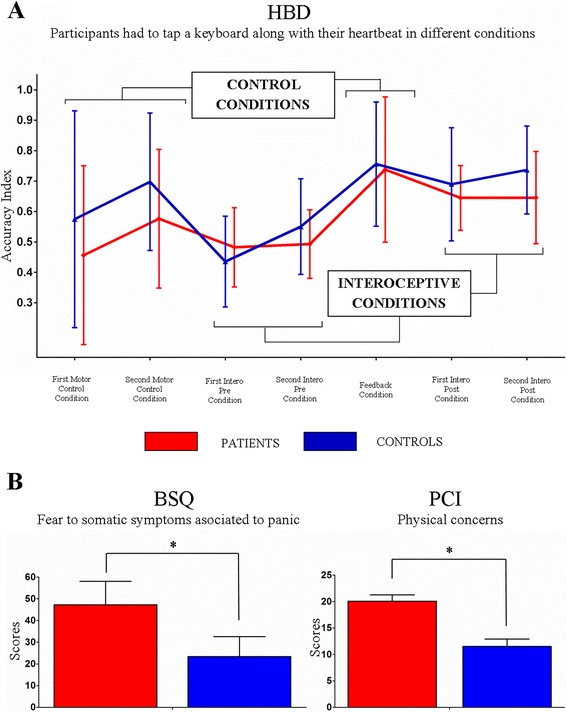
**Interoceptive sensitivity (IS): (A)** Heartbeat Detection Task (HBD). The Accuracy Index can vary between 0 and 1, with higher scores indicating better accuracy. No differences were found between groups in any condition. **Metacognitive interoception (MI): (B)** The BSQ indexes the level of worry about body sensations and the PCI assesses cognitions about threatening impact of anxiety bodily symptoms. Both questionnaires yielded significant differences between groups. Vertical bars indicate standard deviations and asterisks signal significant differences.

There was no effect of group *[F*(1, 26) = 1.76*, p =* 0.19*, ηp2* = 0.06*]* and no interaction between condition and group *[F*(6, 156) = 0.82*, p =* 0.55*, ηp2 =* 0.03*]*. Only an expected [21] and irrelevant effect of condition was observed (Figure 2A and Additional file 1: 3.1). Furthermore, ANCOVA results revealed no differences between interoceptive conditions across groups (Additional file 1: 3.1). No significant differences were found in terms of heart rate (HR) and HRV heart rate variability (HRV) (Additional file 1: 3.5).

### Metacognitive interoception (MI)

Relative to controls, the PA group exhibited higher fear to physical symptoms [BSQ: *F*(1, 30) *=* 42.74*, p <* 0.01*, η*_*p*_^*2*^ *=* 0.58] and higher body anxiety sensations [PCI: *F*(1, 30) = 23.79*, p <* 0.01*, η*_*p*_^*2*^ *=* 0.44] (Figure 2B).

## Discussion

We found no differences in IS between patients and controls. Also, we found evidence for altered MI in patients, who exhibited more worries and catastrophic thoughts about somatic anxiety symptoms.

Previous results of IS in panic populations are inconclusive. They suggest that (i) increased IS is not restricted to panic disorders; (ii) only a small group of patients can be categorized as ‘good heart rate perceivers’, and (iii) results seem to depend on the paradigm used [5,22]. We selected a novel motor resting HBD task that addresses certain methodological limitations of previous reports, such as working memory load and external stimulus interference during interception (Additional file 1: 2.2). Our findings are consistent with the negative results regarding IS [6]. The mental tracking paradigm is the only procedure that has yielded significant differences [2,5], albeit inconsistently. Moreover, accurate heartbeat perception, as measured with this paradigm, is uncommon in both controls and panic patients [6]. Even with the advantages of our new method, no differences were found regarding IS. In addition, the inclusion of BDI and STAI scores as co-factors suggests that these negative results are not affected by such measures (Additional file 1: 3.1 and 3.6).

Together with previous research, our results suggest that this bottom-up process could be a vulnerability factor, but not a pivotal one in the pathogenesis of panic [5].

Regarding MI, our results showed that patients have more worries about body sensations than controls. This is consistent with previous research [6,9,10] and with cognitive models of panic suggesting that the misinterpretation of body signals is a risk factor for panic attacks [8]. In addition, recent prediction coding models of anxiety [23] propose that ‘interoceptive prediction schemas’ (beliefs and predictions about bodily sensations) produce inaccurate predictions about body signals. Moreover, panic treatments based on the modification of biased threatening beliefs about body symptoms are the most effective ones [24]. Thus, the modification of beliefs about the threatening value of bodily sensations might be a fundamental mechanism underlying effectiveness of cognitive interventions.

In conclusion, the present findings suggest differential contributions of IS and MI to panic attacks. This distinction aligns well with reports showing that both dimensions are not associated [3]. The absence of such correlations in our data corroborates such results (Additional file 1: 3.4).

Two limitations in the present study are its small sample size and the diagnostic variability among patients. Nevertheless, we have reported for the first time the comparison of IS and MI in PA using a more robust HBD paradigm than previous ones. In addition, other studies have found no differences in mixed diagnostic groups [2,9]. Moreover, our results remained the same when considering only patients with panic disorder (see reanalysis in Additional file 1: 3.2). The dissociation between IS and MI suggests that further studies should include a multidimensional interoceptive assessment. Another limitation was that IS was measured at rest. Previous studies have shown increased IS with elevated arousal [9,22]. However, our goal was to determine whether classical findings during such a state would be replicated given the demands of our design. Finally, the high proportion of patients under medication could be considered a limitation. Nonetheless, a single-case analysis revealed no effect of medication on IS (Additional file 1: 3.7).

## Conclusion

Significant differences were observed only in the beliefs that patients have about somatic sensations but not in their sensitivity to detect them. Considering these results, previous studies, and anxiety models, it seems that IS might be a vulnerability factor for panic attacks. Still, the fundamental mechanism in the pathogenesis of panic attacks might be a tendency to experience somatic/body signals as threatening sensations.

## Additional file

Additional file 1:
**Additional Methods and Results.** In this additional file we provide the following information about: 1) previous studies regarding interoception and panic; 2) a further clinical description of the patients’ sample; 3) a detailed description of the Motor Heartbeat Detection Task (HBD) and 4) additional results and conclusions.

## Abbreviations

(IS): Interoceptive sensitivity
(MI): Metacognitive interoception
(HBD): Heartbeat detection task
(PA): Anxiety patients that experienced at least one panic attack episode
(SCID-I): Structured Clinical Interview for DSM-IV
(BDI): Beck’s Depression Inventory
(STAI): State Trait Anxiety Inventory
(BSQ): The Body Symptoms Questionnaire
(ACQ): Agoraphobic Cognitions Questionnaire
(PCI): Physical Concern Index
