# Supplementary material for: The roles of interoceptive sensitivity and metacognitive interoception in panic
Source: Behav Brain Funct. 2015 Apr 8;11:14. doi: 10.1186/s12993-015-0058-8 (PMC4422149; doi:10.1186/s12993-015-0058-8)
Supplement: Additional file 1: — Additional Methods and Results. In this additional file we provide the following information about: 1) previous studies regarding interoception and panic; 2) a further clinical description of the patients’ sample; 3) a detailed description of the Motor Heartbeat Detection Task (HBD) and 4) additional results and conclusions. [file 12993_2015_58_MOESM1_ESM.doc]

**Additional File 1.**

**Summary:**

1. **Previous results and sample analysis.**
   1. Table 1. Inconclusive results about IS in panic disorder vs others anxiety disorders studies.
   2. Table 2: Patients Sample: medication, frequently of panic attacks, comorbidity
2. **Motor Heartbeat Detection Task (HBD)**
   1. Experimental design.
   2. Advantages of the motor HBD task.
   3. Procedure.
3. **Additional results and conclusions**
   1. HBD additional results.
   2. PD patients vs controls.
   3. Influence of feedback in interoceptive sensitivity.
   4. IS and MI correlations
   5. HR and HRV results
   6. Influence of anxiety and depression on interoception
   7. Influence of medication on IS.

**1. Previous results and sample analysis.**

- 1. **Table 1. Inconclusive results about IS in panic disorder vs others anxiety disorders** studies.

| Authors | Participants | Method | IS Results |
| --- | --- | --- | --- |
| Ehlers and Breuer (1992) (study one) | panic disorder (N= 65) infrequent panic (N= 50)  simple phobia (N= 27)  normal control (N = 46) | Mental tracking (Schandry) | PD > others groups. |
| Ehlers and Breuer (1992) (study two) | panic disorder (N= 13)  GAD (n = 15)  major depression (N= 16) | Mental tracking (Schandry) | PD = GAD> MD |
| Anthony, et al. (1995) | panic disorder (N= 20)  social phobia (N= 20)  normal control (N = 20) | Mental tracking (Schandry)  (pre and post exercise) | No differences physical exercise improved accuracy in all groups |
| Van der Does et al. (1997) | panic disorder (N= 23)  major depression (N= 16)  normal control (N = 21) | Mental tracking (Schandry) | No differences |
| Zoellner and Craske (1999) | infrequent panic attacks (N=31)  normal control (N=27) | Mental tracking (Schandry) | iPA> controls |
| Barsky et al. (1994) | panic disorder (N= 32)  palpitations, no PD (N= 99)  normal control (N = 64) | Discrimination Task | No differences |
| Richards et al (1996) | panic disorder (N=26)  normal control (N=14) | Discrimination Task | Relaxation: No differences |
| Van der Does et al. (2000) | Seven studies (N=709) | Meta-analisis | PD> MD> iPA> normal controls |

PD: panic disorder; GAD: generalized anxiety disorder; MD: major depression; iPA: infrequent panic attack.

**1.2 Table 2: Patients s**ample: medication, frequently of panic attacks, comorbidity

| **Sample characteristics** | **Number of subjects** |
| --- | --- |
| Medication*:   - Citalopram - Sertraline - Atenolol - Venlafaxine - Clonazepam | 1  2  1  1  6 |
| Frequently of panic attacks   - One or more per day - One or more per week - One or more per moth - One or more in per year | 0  11  14  21 |
| Comorbidity   - Panic disorder –PTSD | 1 |

* = generic name of the drug // PTSD= post-traumatic stress disorder.

**2. Motor Heartbeat Detection Task (HBD)**

**2.1. Experimental design.**

We carried out a behavioral HBD that has already been validated and used in previous works of our group . In this task, participants had to tap a key on a computer keyboard along with their heartbeats in different conditions during resting state. We have not manipulated the participants’ arousal levels during the task. Although previous studies have found IS differences relative to higher arousal levels , we have chosen to assess IS at rest as our aim was to explore the patients’ performance in a novel interoceptive task during conditions comparable to other classical resting IS paradigms (mental tracking and discrimination tasks) . The latter have yielded inconclusive results of IS in panic populations.

We asked all subjects to perform the task with the index finger of their dominant hand. During a first (motor) control condition, participants were instructed to follow two audio recordings of sampled heartbeats. The first one featured beats at a constant frequency (60 bpm), while the second one presented beats manipulated to have an inconstant frequency. Next, they were instructed to follow their own heartbeats with no external stimulation or feedback in two conditions, called first and second intero-pre condition, respectively. Then, in the feedback-motor condition, they were requested to do the same while receiving simultaneous auditory feedback of their own heart provided through an online EKG signal. Finally, in the first and second intero-post conditions, they were once again told to follow their own heartbeat without any feedback, twice. These seven conditions provide us with a measure of merely audio-motoric performance (first and second conditions), as well as with a cardiac interoceptive measure (intero pre- and post- conditions), previous to and after the feedback condition. Thus, the subjects were able to implicitly compared their performance in the intero pre-conditions against this auditory cue and, consequently, enhanced their performance according to this feedback during the intero post-conditions. Thus, our experimental design has two non-interoceptive control conditions: motor and feedback condition. In lights of previous reports with this HBD task , we did not expect to find differences in either of these conditions. The emergence of significant differences would indicate subjects that were not following instructions.

ECG signal was recorded with an ad-hoc circuit composed of an amplifier AD620 and a band-pass filter (low 0.05 Hz, high 40 Hz) and then analogically fed to a laptop computer’s audio-card. Three Ag/Ag-Cl adhesive electrodes were placed to every participant in lead-II positions, together with headphones for audio stimuli delivery. The signal was processed on-line with a PsychToolbox script running on Matlab platform (MathWorks)

Each block lasted two minutes. The total length of the task was 20-25 minutes (including instructions time).

In order to measure ability of participants to follow their heartbeats, we calculated an Accuracy Index based on the following intermediate outputs:

a) Total Correct Answers: shows the total number of each of the subject’s responses that correspond to his/her own heartbeat. Every motor response is compared within a specific time window following every recorded R-wave; if the tap input is temporally locked within a time window for any heartbeat, that response is considered as correct (the time window is determined by the subjects’ heart rate: 750 msec after the beat, for a heart rate (HR) less than 69.76; 600 after, for HR between 69.75 and 94.25; and 400 msec after, for HR higher than 94.25). The index is defined as the total sum of all the subject’s responses that fulfill this temporal criterion.

b) Recorded Heartbeats: refers to the total amount of heartbeats recorded in each condition.

Our accuracy index is a modified equation of the one proposed by Schandry for his heartbeat mental tracking method . Schandry uses the total amount of mental heartbeats counted and the total number of heartbeats recorded. As we can discriminate from the total subjects’ responses, the ones that are correct (according to the criterion explained in the first index), we used this more specific measure of IS instead of the total sum of responses. Another difference with Schandry is that we calculated this index for every condition of the task. The accuracy equation we used is:

**1 - (Recorded heartbeats – ∑ Correct Answers)**

**______________________________________**

**Recorded heartbeats**

This interoceptive score can vary between 0 and 1, with higher scores indicating only small differences between correct answers and recorded heartbeats and thus better interoceptive performance. This score could be either positive or negative based on the tendency of participants to overestimate or underestimate their heartbeats. If this index is positive, it indicates that the participants' estimation of heartbeats is lower than recorded heartbeats. Conversely, if negative, it represents a higher estimation of heartbeats compared to the ones recorded.

**2.2- Advantages of the motor HBD task**

In our study, we have employed this new method to measure IS because it presents several methodological advantages regarding other HBD tasks widely used in the anxiety literature (as the mental tracking and heartbeat discrimination paradigms)

First, motor tracking designs have the advantage of avoiding the attentional and working memory loads given that participants not have to retain online a number count to follow their heartbeats. Instead, both mental tracking and discrimination paradigms have a higher demand of complex cognitive processes. In the former one, this burden is given by subjects' mental count of numbers, to keep the track of heartbeats. In the discrimination paradigms, participants have to divide their attention toward their own heartbeats and also toward an external stimuli, with the objective of judge their synchronicity. This external stimulus interference affects participants performance during the HBD task . By releasing subjects from the cognitive overload of complex processes, our methodology allows a more accurate measure of the ability to follow heartbeats sensations.

Second, our method allows us to record each subject's answers and, therefore, we are able to separate which of them are synchronized with heartbeats and which not. This enables us to calculate an accuracy index that reflects participants' performance based on the ratio between correct responses and the total amount of heartbeats recorded.

Finally, the inclusion of a feedback condition provides information about the interoceptive learning skills, which is observed in the performance enhancement of intero-post conditions compared to the intero-pre conditions.

In conclusion, the motor tracking HBD employed in this study presents several advantages regarding the precise measurement of cardiac perception, which overcome limitations of the other abovementioned HBD paradigms.

**2.3- Procedure**

The patients' diagnoses were established through a psychiatric examination carried out by an expert in anxiety disorders (R.K).Next, in a different session, patients and controls were individually assessed with the HBD task. All of the evaluations took place in a noise-free and comfortable environment. Additionaly, in the same session, we administered the self-report questionnaires (BDI, STAI State/Trait, BSQ and PCi). Regarding the “state part” of the STAI, participants completed it as soon as they finished the HBD task. The instruction was. "Take this quiz according to how you felt during the test (referring to the HBD task).

**3. Additional results**

**3.1. HBD additional results**

In HBD, significant differences were found in the Total Accuracy Index when comparing seven conditions interactions within subjects [F(6, 156)=10.50, p<0.01, ηp2= 0.28]. A post-hoc comparison (Tuckey HSD test, MS=0.3; df= 156) revealed that: (i) the feedback condition presented higher scores compared to the first motor condition (p < 0.01) and also compared to both interoceptive pre-conditions (both ps < 0.01); and (ii) both interoceptive post-conditions presented higher scores than the first and second interoceptive pre-conditions (p < 0.05).

In light of a previous study , the subjects’ performance was expected to vary between conditions due to the changes in task settings,. Indeed, participants adjusted their performance in the feedback condition compared to the first and second interoceptive conditions according to available auditory cues. This is illustrated in Supplementary Figure 1: in the feedback conditions the proportion of correct responses between 0.2 and 0.4 milliseconds is notably higher than in the other windows of this condition. Conversely, both the intero pre- and post- conditions presented a distribution of correct responses that is more homogeneous across time windows (except for the last one). This suggests that participants firmly followed their heartbeats during the feedback condition compared to the interoceptive conditions.

Suppl. Figure 1: Subjects’ response distribution


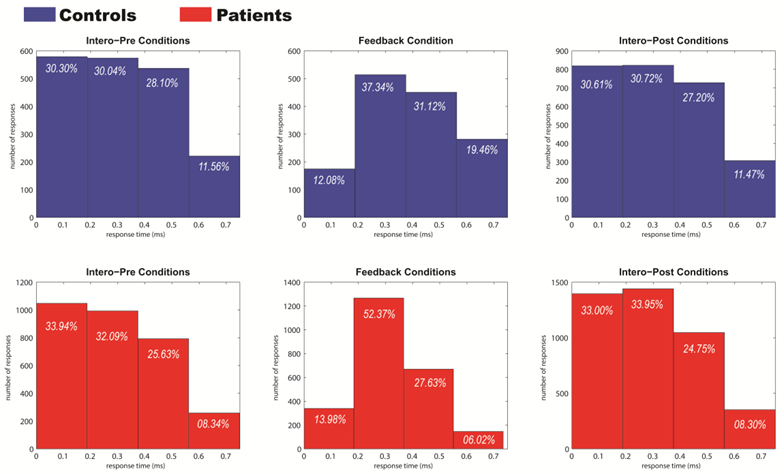


*Suppl. Figure 1: Proportion of correct responses within each time window.*

On the other hand, to overcome the possible influence of anxiety and depression (STAI-S/T & BDI scores) variables on HBD accuracy, we applied a co-variance analysis (ANCOVA) for both intero pre and intero post conditions. No group effects were observed for either condition between samples: First Intero Pre Condition [F(1, 26) <0.01, p =0.96, ηp2 <0.01]; Second Intero Pre Condition [F(1, 27) =1.68, p =0.20, ηp2 =0.06]; First Intero Post Condition [F(1, 27) =0.95, p =0.34, ηp2 =0.03]and Second Intero Post Condition [F(1, 27) =0.00, p =1, ηp2 <0.01].

We also applied co-variance analysis for MI considering the same variables as covariates. Relative to controls, the PA group exhibited higher fear to physical symptoms [BSQ: *F*(1, 25)*=*28.39*, p<*0.01*, η_p_^2^=*0.53] and higher body anxiety sensations [PCI: *F*(1, 25)=12.04*, p<*0.01*, η_p_^2^=*0.32]

**3.2. PD patients vs controls.**

Thirteen subjects of our sample were diagnosed with Panic Disorder (PD), using DSM-IV criteria . With the aim of overcoming the mixture of anxiety diagnostics of our original sample, we compared this subgroup of PD with controls. ANOVA analysis showed similar results than our previous findings.

**Demographic results**

No differences were found in gender [χ2 (1, N = 26)= 0.65, p= 0.42], age [F (1, 24)= 0.25, p= 0.61, η_p_^2^ 0= 0.01], formal education [F (1, 24)= 0.23, p= 0.63, η_p_^2^ < 0.01] or body mass index [F (1, 24)= 1.12, p= 0.29, η_p_^2^ = 0.04] between groups.

**Clinical results**

We observed a significant difference for BDI score between groups [F (1, 23)= 12.91, p< 0.01, η_p_^2^= 0.35], which revealed higher scores of depressive symptoms in patients compared to controls. We did not observe between group differences for STAI-State subscale [F (1, 23)= 1.06, p= 0.31, η_p_^2^= 0.04]. However, significant differences for STAI-Trait subscale [F (1, 24)= 12.19, p< 0.05, η_p_^2^= 0.33] were observed. These showed that patients had significantly higher anxiety scores than controls.

**Interoceptive sensitivity results**

No group effects were observed [F (1, 19)= 2.84, p=0.11, ηp2=0.13]. Although a significant condition x group interaction [F (6, 114)= 3.2, p< 0.01, ηp2= 0.14] was observed, this result was supported by the inter-conditions comparison (e.g., motor conditions of the control group vs. interoceptive conditions of the patient group). Performance in the HBD task in both samples yielded higher scores in the control conditions (motor and feedback) and lower scores in the interoceptive conditions (with post-interocetive conditions showing higher values than the pre-interoceptive conditions). These higher and lower scores between conditions were the reason why the group effect reached significance in this new analysis. Moreover, if we analyze the post-hoc comparison (Tukey HSD test, MS=0.04; df= 108.63), we corroborate that group comparisons within the same conditions (e.g. intero pre-condition of the control group vs. intero pre-condition of the patient group) showed no significant difference in any conditions (p > 0.05).

**Metacognitive interoception results**

The BSQ score showed that patients had significantly higher scores about their fear to physical symptoms than controls [F (1, 14)= 11.659, p< 0.01, η_p_^2^= 0.04]. In the same way, the PCL showed differences between groups [F (1, 14)= 36.31, p< 0.01, η_p_^2^= 0.72] showing that body anxiety sensations were more threatening for the group of patients.

**3.3. Influence of feedback in interoceptive sensitivity.**

Results shown in the previous section regarding the interaction between pre- and post- interoceptive conditions demonstrated a clear effect of feedback in participants' interoceptive performance (Additional Results 3.1). Both post-interoceptive conditions presented significantly higher accuracy scores compared to the conditions before the feedback.

An unpublished study of our group, whit the same HBD task, has shown that the heartbeat evoked potential (known as HEP, an ERP signature of cortical cardiac processing ), was higher in participants who enhanced their interoceptive sensitivity accuracy after feedback compared to the ones that had a similar performance in both pre- and post-interoceptive conditions . This might suggest that "interoceptive learners" have extracted some relevant sensory and perceptual information from the feedback condition that allowed them to perform better in the post-conditions.

On the other hand, a recent study highlights the possible influence of belief about heart rate regarding the effects of feedback . The authors demonstrated that knowing about heart rate may determine accurate heartbeat counting, regardless of any training or learning effect. Accordingly, they have suggested that the enhancement during post-interoceptive conditions might reflect the knowledge about heart rate frequency that participants get from the feedback condition, rather than signal unmasking. Nevertheless, this finding is not supported by electrophysiological evidence; hence, although the influence of heart rate belief was not assessed in the unpublished work of our group , it is unlikely that this information from the feedback condition could have enhanced the HEP in "interoceptive learners" compared to "non-learners", without unmasking some relevant sensory or perceptual information.

Despite the consideration of the effect of feedback as an unmasking effect or just as a source of information, we compared the interoceptive conditions pre- and post- feedback to assess differences between groups. First, we calculated the average of the intero-pre condition scores. Then, we calculated the average from the intero-post conditions. Finally, we performed a subtraction of these two averages (inter-pre average minus intero-pos average). This new index was used in a one way ANOVA between groups. No significant differences were found: [F (1, 32)=2.34, p=0.14, ηp2=0.07]. This result suggests that both samples had similar skills to integrate external stimuli with interoceptive (visceral) information.

**3.4. IS and MI: correlations.**

Pearsons´s correlation test was used. We selected two indexes of IS (average of the two intero-pre conditions and the average of the other two intero-post conditions) and both indexes of MI (BSQ and PCL). No significant correlations were found between IS and MI in the entire sample [intero-pre conditions average-BSQ: r= 0.02, p=0.92; intero-pre conditions average-PCL: r= 0.22, p=0.32; intero-post conditions average-BSQ: p= -0.28, p=0.19; intero-post conditions average-PCL: r= -0.05, p=0.80].

**3.5 Heart-rate and heart-rate variability analysis**

**Introduction**

Given the possible influence of cardiodynamics variables on interoceptive sensitivity performance, we compared heart rate (HR) and heart rate variability (HRV) between groups. Our results showed that groups presented similar HR and HRV measures during interoceptive task, discarding the role of these variables as confounders.

Data analysis

The ECG signal recorded from each condition (with the exception of the motor one) was utilized to calculate heart rate and heart-rate variability. We imported beat-to-beat RR interval data (extracted from the ECG using Matlab platform) to the Kubios HRV , an advance software for heart rate variability (HRV) analysis. This software automatically analyzed the HRV in both time and frequency domains. Given that the analysis was done for short times (2.5 min approx. for each condition), an autoregressive (AR) algorithm was used to calculate power spectral, accordance to previous recommendations . This algorithm generates a power spectral analysis with different frequency bands: high frequency (HF), low frequency (LF) and very low frequency (VLF). The HF component has been associated with the respiratory rhythm of heart period variability and is considered a marker of vagal modulation . The LF component reflects the rhythm corresponding to vasomotor waves present in heart period and arterial pressure variability and is a marker of sympathetic modulation . The LF/HF ratio has been suggested to mirror sympatho/vagal balance . We expressed these frequency components in normalized units (n.u.) that represent the relative value of each power component in proportion to the total power minus the VLF component .

The Kubios HRV also gives a mean heart rate (HR) measure for each condition. ANOVA test was performed for HR and HRV only comparing the interoceptive conditions (the average of both pre- and post-feedback conditions).

**Results**

The statistical analysis of the HRV was performed on normalized units (n.u.) from the LF/HF ratio because this is a unique measure that allows comparing the similarity of sympatho/vagal balances from different samples.

Regarding HRV, no differences were found between groups in either the intero-pre conditions [F(1, 32) =3.19, p =0.08, ηp^2^ =0.09] and the intero-post conditions [F(1, 32) =1.86, p =0.18, ηp^2^ =0.05]. The same was true for the HR analysis: intero-pre conditions [F(1, 32) =0.81, p =0.37, ηp^2^ =0.02]; and intero-post conditions [F(1, 32) =0.56, p =0.45, ηp^2^ =0.01].

Table 3.4. Mean and SD in HR and HRV between groups.

| *Groups* | *Heart Rate (HR)* | |
| --- | --- | --- |
|  | *Intero-pre Conditions* | *Intero-post Conditions* |
| *Patients* | *M= 79.79 (14.68)* | *M= 78.49 (13.99)* |
| *Controls* | *M= 74.86 (16.60)* | *M= 74.92 (12.30)* |
|  | *Heart Rate Variability (HRV)* | |
| *Patients* | *M= 2.35 (1.77)* | *M= 2.39 (1.85)* |
| *Controls* | *M= 5.39 (7.53)* | *M= 7.14 (15.93)* |

*M =average;(standard deviation)*

**Discussion**

Both HR and HRV were measured due to their possible influence on IS.

HR and HRV results showed no differences between groups and conditions. Thus these variables seem not to have biased interoceptive results.

**3.6. Influence of anxiety and depression on interoception.**

Several studies have analyzed the associations of mood and anxiety with interoceptive sensitivity (IS) [see the reviews from Domschke, K. et.al (2010) and Harshaw, C. (2014) ]. This topic has been evaluated in the mainstream literature using both healthy subjects and self-report questionnaires about anxiety and mood levels, as well as clinical samples.

We used two measures to assess anxiety: state and trait scores obtained from the STAI questionnaire . Trait anxiety represents a stable general tendency to respond to threats with anxiety, whereas state anxiety refers to a transitory emotional condition, which intensity fluctuates over time.

Previous results about the relationship between these two measures and interoception are ambiguous and come mainly from research with non-clinical populations. Relative to state anxiety, the majority of research has shown that higher scores in this measure are positively associated with accuracy in IS . However, these results were based on non-clinical populations. One study with panic disorder patients found the same positive correlation (considering the whole group) , whereas another one failed to find associations using a similar clinical population . On the other hand, trait anxiety has yielded more consistent results about its positive association with IS , but these findings were also based on samples without an anxiety diagnosis.

The evidence regarding the association between depression and interoceptive dysfunction is more consistent than in anxiety research . Several studies have shown a decreased IS in patients with a major depression diagnosis as well as in healthy subjects with high scores in mood questionnaires (BDI) .

Considering this background, anxiety and mood levels (from the STAI and BDI questionnaires) were used as co-factors in an ANCOVA for evaluating both IS and MI. This approach was further justified since our clinical sample obtained significantly higher scores in these measures than did the controls (with the exception of state anxiety). We did not conduct correlation analyses between interoception and anxiety and mood levels because this was not a relevan approach to address our hypotheses and main questions. As stated before, findings in anxiety are inconclusive and our interest was focused on the possible differences between a well characterized clinical sample and a healthy control group. In this way, our negative results suggest that IS is not a pivotal factor in the pathogenesis of panic, whereas MI might represent a fundamental mechanism underlying panic disorders. The relevance of these results is that they are supported under the influence of the anxiety and mood scores form the self-report questionnaires.

**3.7. Influence of medication on IS.**

All patients were first evaluated by expert psychiatrists and psychologists from the anxiety clinic of INECO. Some patients that were in an acute panic state at admission were medicated. In this way, despite the medication, we included them in our study given that they were suffering several panic attacks and fulfilled with inclusion criteria.

Almost half of the patients (47%) were under medication. To corroborate our findings and test for the influence of medication, we used a modified two-tailed t-test for single subject comparison . This statistical approach has already been used with this HBD task in previous studies of our group and in other papers . This methodology allows the assessment of significance by comparing multiple individual’s test scores with norms derived from small samples. Although parametric statistics usually requires comparison groups of about 30 subjects, the one-tailed t-test allows the assessment of significance by comparing an individual’s score to the scores obtained in a small control sample (with even fewer than 5 subjects) . Using this modified t-test, we compared each anxiety patient against the control sample in all the interoceptive conditions (pre- and post- feedback). Table 4 summarizes the main effects observed in the different interoceptive conditions when comparing patients against controls.”

Table 4. Crawford t-test for single subject comparison.

|  | FIRST INTERO PRE CONDITION | | | SECOND INTERO PRE CONDITION | | | FIRST INTERO POST CONDITION | | | SECOND INTERO POST CONDITION | | |
| --- | --- | --- | --- | --- | --- | --- | --- | --- | --- | --- | --- | --- |
| PATIENTS | t | P | Zcc | t | P | Zcc | t | p | Zcc | t | p | Zcc |
| 1 | -0,08 | 0,94 | -0,08 | -0,36 | 0,73 | -0,37 | -0,50 | 0,63 | -0,52 | -1,32 | 0,21 | -1,37 |
| 2 | 0,11 | 0,91 | 0,12 | -0,44 | 0,67 | -0,46 | 0,16 | 0,87 | 0,17 | -0,51 | 0,62 | -0,53 |
| 3 | -0,05 | 0,96 | -0,05 | -0,17 | 0,87 | -0,18 | 0,40 | 0,70 | 0,42 | 1,14 | 0,28 | 1,19 |
| 4 | 0,97 | 0,35 | 1,01 | 0,57 | 0,58 | 0,59 | 1,05 | 0,32 | 1,08 | 1,04 | 0,32 | 1,08 |
| 5 | -0,25 | 0,81 | -0,26 | -1,07 | 0,30 | -1,11 | -0,30 | 0,77 | -0,31 | -0,02 | 0,99 | -0,02 |
| 6 | 0,23 | 0,82 | 0,24 | -0,97 | 0,35 | -1,00 | -1,40 | 0,19 | -1,46 | **-2,58** | **0,02*** | **-2,68** |
| 7 | 1,18 | 0,26 | 1,23 | -0,43 | 0,67 | -0,45 | 0,10 | 0,93 | 0,10 | -0,54 | 0,60 | -0,56 |
| 8 | -0,26 | 0,80 | -0,27 | -0,13 | 0,90 | -0,14 | 0,40 | 0,70 | 0,41 | -0,15 | 0,88 | -0,15 |
| 9 | 0,62 | 0,55 | 0,64 | 0,03 | 0,97 | 0,03 | -0,60 | 0,56 | -0,62 | -2,02 | 0,07 | -2,10 |
| 10 | -0,41 | 0,69 | -0,42 | -0,02 | 0,98 | -0,02 | 0,82 | 0,43 | 0,85 | -1,26 | 0,23 | -1,31 |
| 11 | **2,36** | **0,04*** | **2,44** | 0,89 | 0,39 | 0,92 | 0,54 | 0,60 | 0,56 | 1,19 | 0,26 | 1,23 |
| 12 | **2,37** | **0,04*** | **2,46** | 1,45 | 0,17 | 1,50 | -0,13 | 0,90 | -0,14 | -0,19 | 0,85 | -0,20 |
| 13 | -0,14 | 0,89 | -0,15 | -0,92 | 0,38 | -0,96 | 0,00 | 1,00 | 0,00 | 0,05 | 0,96 | 0,06 |
| 14 | 0,97 | 0,35 | 1,01 | -0,36 | 0,72 | -0,38 | -0,56 | 0,59 | -0,58 | -1,46 | 0,17 | -1,51 |
| 15 | 1,58 | 0,14 | 1,64 | 0,75 | 0,47 | 0,78 | 0,87 | 0,40 | 0,91 | 0,93 | 0,37 | 0,96 |
| 16 | 0,74 | 0,47 | 0,77 | 0,44 | 0,67 | 0,45 | 0,34 | 0,74 | 0,35 | -0,56 | 0,58 | -0,58 |
| 17 | 1,08 | 0,30 | 1,12 | 0,56 | 0,58 | 0,58 | -0,15 | 0,88 | -0,16 | 0,71 | 0,49 | 0,74 |
| 18 | MD | MD | MD | 0,61 | 0,55 | 0,63 | 0,32 | 0,75 | 0,34 | -0,52 | 0,61 | -0,54 |
| 19 | -0,30 | 0,77 | -0,31 | -0,67 | 0,52 | -0,70 | -0,70 | 0,50 | -0,72 | -0,74 | 0,47 | -0,77 |
| 20 | 1,71 | 0,11 | 1,77 | 0,01 | 0,99 | 0,01 | 0,40 | 0,70 | 0,41 | 0,86 | 0,41 | 0,89 |
| 21 | -0,61 | 0,55 | -0,63 | -1,75 | 0,11 | -1,81 | 0,45 | 0,66 | 0,47 | 0,23 | 0,82 | 0,24 |

*MD = missing data; * significant differences.*

As seen in the table, only two subjects presented differences in the first intero pre condition, and one in the second intero post condition. These results support the robustness of our data: even when applying a more rigorous statistical analysis, we found differences only in a very small percentage of the sample (less than 4%), which is not even constant across conditions. Thus, if patients had actually presented higher interoceptive sensitivity than controls (masked by the influence on medication), the single-case approach would have revealed a larger number of significant differences. These differences would represent the number of patients who were not under medication and had higher IS accuracy than controls. Notwithstanding, the possibility remains that only the medicated patients exhibited better IS performance. However, in light of previous studies that have demonstrated that only a small group of patients can be categorized as 'good heart-rate perceivers', it is quite unlikely that all of them had been in the medicated sub-sample. And, if so, they would represent a small proportion of the total group, not representative enough of anxiety disorders. In conclusion, this single-case analysis allows us to discard the influence of medication in patients’ performance as a confound.

**References:**

1. Ehlers A, Breuer P: **Increased cardiac awareness in panic disorder.** *Journal of abnormal psychology* 1992, **101:**371-382.

2. Antony M, Brown, T., Craske, M., Barlow, D., Mitchell, W., & Meadows, E.: **Accuracy of heartbeat perception in panic disorder, social phobia, and nonanxious subjects.** *Journal of Anxiety Disorders* 1995, **9:**355–371.

3. Van der Does WAJ, van Dyck, D. R., & Spinhoven, P.: **Accurate heartbeat perception in panic disorder: Fact and artefact.** *Journal of Affective Disorders* 1997, **43:**121–130.

4. Zoellner LA, Craske MG: **Interoceptive accuracy and panic.** *Behaviour research and therapy* 1999, **37:**1141-1158.

5. Barsky AJ, Cleary PD, Sarnie MK, Ruskin JN: **Panic disorder, palpitations, and the awareness of cardiac activity.** *The Journal of nervous and mental disease* 1994, **182:**63-71.

6. Richards JCE, Lorraine V.: **Cardiac acuity in panic disorder.** *Cognitive Therapy & Research* 1996, **20:**361.

7. Van der Does A, Antony M, Ehlers A, Barsky A: **Heartbeat perception in panic disorder: a reanalysis.** *Behaviour research and therapy* 2000, **38:**47-62.

8. Couto B, Salles A, Sedeno L, Peradejordi M, Barttfeld P, Canales-Johnson A, Dos Santos YV, Huepe D, Bekinschtein T, Sigman M, et al: **The man who feels two hearts: the different pathways of interoception.** *Soc Cogn Affect Neurosci* 2014, **9:**1253-1260.

9. Melloni M, Sedeno L, Couto B, Reynoso M, Gelormini C, Favaloro R, Canales-Johnson A, Sigman M, Manes F, Ibanez A: **Preliminary evidence about the effects of meditation on interoceptive sensitivity and social cognition.** *Behavioral and brain functions : BBF* 2013, **9:**47.

10. Sedeno L, Couto B, Melloni M, Canales-Johnson A, Yoris A, Baez S, Esteves S, Velasquez M, Barttfeld P, Sigman M, et al: **How do you feel when you can't feel your body? Interoception, functional connectivity and emotional processing in depersonalization-derealization disorder.** *PloS one* 2014, **9:**e98769.

11. Zoellner L, Craske M: **Interoceptive Accuracy and Panic.** *Behaviour research and therapy* 1999, **37:**1141-1158.

12. Couto B, Salles A, Sedeño L, Peradejordi M, Barttfeld P, Canales-Johnson A, Dos Santos YV, Huepe D, Bekinschtein T, Sigman M, et al: **The man who feels two hearts: Heartbeat detection, social cognition and emotional processing through different interoceptive pathways. In press.** *Soc Cogn Affect Neurosci* 2013.

13. DH B: *The Psychophysics Toolbox. Spatial vision 1997, 10:433-43.* 1997.

14. Schandry R: **Heart beat perception and emotional experience.** *Psychophysiology* 1981, **18:**483-488.

15. American Psychiatric Asociation A: *Diagnostic and Statistical Manual of Mental Disorders.* New York: Masson; 1994.

16. Montoya P, Schandry R, Muller A: **Heartbeat evoked potentials (HEP): topography and influence of cardiac awareness and focus of attention.** *Electroencephalography and clinical neurophysiology* 1993, **88:**163-172.

17. Bekinschtein TA C-JA, Silva C, Huepe D, Rivera-Rei A, Noreika V, Del Carmen Garcia M, Silva W, Sedeño L, Kargieman L, Baglivo F, Chennu S, Ibanez A and Rodriguez EF: **Learn from your heart: dissociable neural markers for objective interoceptive performance and metacognitive awareness in auditory feedback.** In *Book Learn from your heart: dissociable neural markers for objective interoceptive performance and metacognitive awareness in auditory feedback* (Editor ed.^eds.). City; 2013.

18. Ring C, Brener J, Knapp K, Mailloux J: **Effects of heartbeat feedback on beliefs about heart rate and heartbeat counting: A cautionary tale about interoceptive awareness.** *Biological psychology* 2015, **104:**193-198.

19. Tarvainen MP, Niskanen JP, Lipponen JA, Ranta-Aho PO, Karjalainen PA: **Kubios HRV - Heart rate variability analysis software.** *Computer methods and programs in biomedicine* 2013.

20. Taskforce: **Heart rate variability: standards of measurement, physiological interpretation and clinical use. Task Force of the European Society of Cardiology and the North American Society of Pacing and Electrophysiology.** *Circulation* 1996, **93:**1043-1065.

21. Malliani A, Pagani M, Lombardi F, Cerutti S: **Cardiovascular neural regulation explored in the frequency domain.** *Circulation* 1991, **84:**482-492.

22. Eckberg DL: **Sympathovagal balance: a critical appraisal.** *Circulation* 1997, **96:**3224-3232.

23. Pagani M, Lombardi F, Guzzetti S, Rimoldi O, Furlan R, Pizzinelli P, Sandrone G, Malfatto G, Dell'Orto S, Piccaluga E, et al.: **Power spectral analysis of heart rate and arterial pressure variabilities as a marker of sympatho-vagal interaction in man and conscious dog.** *Circulation research* 1986, **59:**178-193.

24. Domschke K, Stevens S, Pfleiderer B, Gerlach AL: **Interoceptive sensitivity in anxiety and anxiety disorders: an overview and integration of neurobiological findings.** *Clinical psychology review* 2010, **30:**1-11.

25. Harshaw C: **Interoceptive Dysfunction: Toward an Integrated Framework for Understanding Somatic and Affective Disturbance in Depression.** *Psychological bulletin* 2014.

26. Spielberger CD, Gorsuch RL, Lushene RE: *Manual for the Stait-Trait Anxiety Inventory.* 1970.

27. Ludwick-Rosenthal R, Neufeld RW: **Heart beat interoception: a study of individual differences.** *International journal of psychophysiology : official journal of the International Organization of Psychophysiology* 1985, **3:**57-65.

28. Naring GW, van der Staak CP: **Perception of heart rate and blood pressure: the role of alexithymia and anxiety.** *Psychotherapy and psychosomatics* 1995, **63:**193-200.

29. Karsdorp PA, Kindt M, Rietveld S, Everaerd W, Mulder BJ: **False heart rate feedback and the perception of heart symptoms in patients with congenital heart disease and anxiety.** *International journal of behavioral medicine* 2009, **16:**81-88.

30. Pollatos O, Gramann K, Schandry R: **Neural systems connecting interoceptive awareness and feelings.** *Human brain mapping* 2007, **28:**9-18.

31. Pollatos O, Herbert BM, Kaufmann C, Auer DP, Schandry R: **Interoceptive awareness, anxiety and cardiovascular reactivity to isometric exercise.** *International journal of psychophysiology : official journal of the International Organization of Psychophysiology* 2007, **65:**167-173.

32. Pollatos O, Traut-Mattausch E, Schandry R: **Differential effects of anxiety and depression on interoceptive accuracy.** *Depression and anxiety* 2009, **26:**167-173.

33. Richards JC, Bertram S: **Anxiety sensitivity, state and trait anxiety, and perception of change in sympathetic nervous system arousal.** *Journal of anxiety disorders* 2000, **14:**413-427.

34. Furman DJ, Waugh CE, Bhattacharjee K, Thompson RJ, Gotlib IH: **Interoceptive awareness, positive affect, and decision making in major depressive disorder.** *J Affect Disord* 2013, **151:**780-785.

35. Terhaar J, Viola FC, Bar KJ, Debener S: **Heartbeat evoked potentials mirror altered body perception in depressed patients.** *Clinical neurophysiology : official journal of the International Federation of Clinical Neurophysiology* 2012, **123:**1950-1957.

36. Dunn BD, Dalgleish T, Ogilvie AD, Lawrence AD: **Heartbeat perception in depression.** *Behaviour research and therapy* 2007, **45:**1921-1930.

37. Herbert BM, Herbert C, Pollatos O: **On the relationship between interoceptive awareness and alexithymia: is interoceptive awareness related to emotional awareness?** *Journal of personality* 2011, **79:**1149-1175.

38. Crawford JR, Garthwaite PH, Howell DC: **On comparing a single case with a control sample: an alternative perspective.** *Neuropsychologia* 2009, **47:**2690-2695.

39. Couto B, Salles A, Sedeno L, Peradejordi M, Barttfeld P, Canales-Johnson A, Dos Santos YV, Huepe D, Bekinschtein T, Sigman M, et al: **The man who feels two hearts: the different pathways of interoception.** *Soc Cogn Affect Neurosci* 2013.

40. Straube T WA, Schmidt S, Raschdorf C, Preul C, et al.: **No impairment of recognition and experience of disgust in a patient with a righthemispheric lesion of the insula and basal ganglia.** *Neuropsychologia* 2010, **48**.

41. DC CJH: **Comparing an individual’s test score against norms derived from small samples.** *Clin Neuropsychol* 1998, **12:**482–486.
